# Breast screening atypia and subsequent development of cancer: protocol for an observational analysis of the Sloane database in England (Sloane atypia cohort study)

**DOI:** 10.1136/bmjopen-2021-058050

**Published:** 2022-01-07

**Authors:** David Jenkinson, Karoline Freeman, Karen Clements, Bridget Hilton, Joanne Dulson-Cox, Olive Kearins, Nigel Stallard, Matthew G Wallis, Nisha Sharma, Cliona Kirwan, Sarah Pinder, Elena Provenzano, Abeer M Shaaban, Hilary Stobart, Samantha McDonnell, Alastair M Thompson, Sian Taylor-Phillips

**Affiliations:** 1 Warwick Medical School, University of Warwick, Coventry, UK; 2 Screening Quality Assurance Services, NHS England and NHS Improvement, Birmingham, UK; 3 Cambridge Breast Unit and NIHR Cambridge Biomedical Research Centre, Cambridge University Hospitals NHS Trust, Cambridge, UK; 4 Breast Screening Unit, Seacroft Hospital, Leeds Teaching Hospitals NHS Trust, Leeds, UK; 5 Department of Surgery, The University of Manchester, Manchester, UK; 6 School of Cancer & Pharmaceutical Sciences, King's College London, London, UK; 7 Comprehensive Cancer Centre at Guy’s Hospital, Guy's and St Thomas' NHS Foundation Trust, London, UK; 8 Histopathology and NIHR Cambridge Biomedical Research Centre, Cambridge University Hospitals NHS Foundation Trust, Cambridge, UK; 9 University Hospitals Birmingham NHS Foundation Trust, Queen Elizabeth Hospital Birmingham, Birmingham, UK; 10 Independent Cancer Patients' Voice, London, UK; 11 Department of Surgical Oncology, Dan L Duncan Comprehensive Cancer Center, Baylor College of Medicine, Houston, Texas, USA

**Keywords:** health policy, protocols & guidelines, breast tumours, public health

## Abstract

**Introduction:**

The National Health Service (NHS) Breast Screening Programme aims to detect cancer earlier when treatment is more effective but can harm women by over diagnosing and overtreating cancers which would never have become symptomatic. As well as breast cancer, a spectrum of atypical epithelial proliferations (atypia) can also be detected as part of screening. This spectrum of changes, while not cancer, may mean that a woman is more likely to develop breast cancer in the future. Follow-up of atypia is not evidence based. We currently do not know which atypia should be detected to avoid future cancer. This study will explore how atypia develops into breast cancer in terms of number of women, time of cancer development, cancer type and severity, and whether this varies for different types of atypia.

**Methods and analysis:**

The Sloane cohort study began in April 2003 with ongoing data collection including atypia diagnosed through screening at screening units in the UK. The database for England has 3645 cases (24 September 2020) of epithelial atypia, with follow-up from 1 to 15 years. The outcomes include subsequent invasive breast cancer and the nature of subsequent cancer. Descriptive statistics will be produced. The observed rates of breast cancer at 1, 3 and 6 years for types of atypia will be reported with CIs, to enable comparison to women in the general population. Time to event methods will be used to describe the time to breast cancer diagnosis for the types of atypia, including flexible parametric modelling if appropriate. Patient representatives from Independent Cancer Patients’ Voice are included at every stage of the research.

**Ethics and dissemination:**

The study has received research ethics approval from the University of Warwick Biomedical and Scientific Research Ethics Committee (BSREC 10/20–21, 8 October 2020), Public Health England office for data release approvals (ODR1718_313) and approval from the English Breast Research Advisory Committee (BSPRAC_031). The findings will be disseminated to breast screening clinicians (via journal publication and conference presentation), to the NHS Breast Screening Programme to update their guidelines on how women with atypia should be followed up, and to the general public.

**Strengths and limitations of this study**

- Large prospective cohort study, April 2003 to present, with long follow-up allows detection of subsequent cancers.
- The Sloane database has standardised reporting and high completion.
- No systematic inclusion or random selection of all incidents of women diagnosed with atypia at screening.
- Observational analysis introduces risk of bias from measured and unmeasured confounding.

## Introduction

### Rationale

In England, breast cancer screening is offered every 3 years to women aged 50–70. In 2018/2019, 2.23 million women were screened within the National Health Service (NHS) Breast Screening Programme (NHSBSP)^1^ and nearly 85 000 screened women (3.8%) were referred for further tests due to a potential abnormality detected at initial screening.^1^ Breast screening aims to decrease mortality by detecting breast cancer earlier. A key harm of breast cancer screening is overdiagnosis of cancer which would never have presented symptomatically in the woman’s lifetime. Around 3000 women each year have breast cancer over diagnosed and are overtreated because of breast screening.^2^ Biopsies in referred women detect a rapidly increasing number of atypical epithelial proliferations (atypia(s)). Atypias are a heterogeneous group of abnormalities, which are not cancer but carry a low but significant rate of associated malignancy, necessitating increased sampling (either surgical or vacuum-assisted needle biopsy) to allow more representative histological examination of the area. Owing to improvements in technology we can now detect many smaller lesions^3^ and more lesions are classified as atypia. Atypia is diagnosed in 5%–10% of core needle biopsies performed as part of the NHSBSP.^4 5^ Detecting these lesions has uncertain benefit because of insufficient evidence on their risk of subsequent development into breast cancer. Furthermore, it is not clear what type of cancers subsequently develop, and only women with faster growing cancer types benefit from more frequent screening. This information is key to optimising follow-up and subsequent screening. There is also huge intercentre variability in amount of atypia detected. There are no current guidelines for follow-up after excision of atypia in England.^6^ Some centres undertake annual surveillance mammography for 5 years, but the NHSBSP is not willing to issue guidance or provide funding for surveillance until there is more research, in particular from analysis of the Sloane atypia data.^6^ This study will undertake the first analysis of the Sloane atypia data. We will report the proportion of women with atypia who develop breast cancer by type of atypia and in what time frame to enable policy-makers to decide which type of atypia should be followed-up after screening, if any, and which women should be provided with annual surveillance mammography.

### Available evidence and the evidence gap

Atypia represents a group of diverse abnormalities, including atypical ductal hyperplasia (ADH) or atypical intraductal epithelial proliferation, flat epithelial atypia (FEA), and lobular in situ neoplasia (LISN: atypical lobular hyperplasia (ALH) and lobular carcinoma in situ (LCIS)). These abnormalities are not malignant themselves, however, cancer can coexist with the lesion,^5 7^ and the presence of atypia has been found to confer a 3 to 4 times increased risk of subsequent breast cancer over time.^8 9^

Pre-2015, information about subsequent risk of breast cancer consequent on a diagnosis of atypia was based on a small series of case control studies.^10^ A 2011 meta-analysis of 238 ADH cases and 135 ALH cases reported that, relative to non-proliferative disease, the risk for women with ALH [OR 5.14, 95% CI (3.52 to 7.52)] may be higher than for women with ADH (OR 2.93, 95% CI (2.16 to 3.97)).^11^ A further meta-analysis in 2015^8^ reported a summary relative risk for atypia in general of 3.93 (95% CI (3.24 to 4.76)), based on 13 studies.

More recently there have been four studies^3 9 12 13^ investigating pure, and three studies^14–16^ investigating mixed, forms of atypia. Studies of pure atypia have limited applicability because they were based on cohorts from the USA where diagnostic and follow-up procedures differ from the UK. Studies investigating mixed forms of atypia were mainly small (282^3^ to 698^16^ women) and heterogeneous, using different length of follow-up and different comparators (women with benign biopsies vs women without benign breast disease). Evidence from the UK is minimal and the only study from elsewhere in Europe, where management, pathology guidelines and quality assurance are similar to the UK, is small with 115 cases.^15^ The published literature suggests a variable risk of malignancy due to presence or absence of atypia,^15^ observation versus excision of atypia,^12^ and due to different atypia subtypes.^14–16^ Risk is three times greater for ipsilateral rather than contralateral cancer, suggesting some atypias are a true precursor rather than a general risk factor for development of invasive disease.^12 17^ The long-term risk of cancer in patients with FEA appears to be lower than other atypical epithelial proliferations and may indeed be negligible and not warrant surveillance mammography at the same frequency as that presently undertaken for ADH and LISN.^3^

The variable risk of malignancy in different series could also be attributable to statistical error due to small sample sizes, chemoprevention in some women in a number of cohort studies, and lack of uniform reporting of benign breast disease between pathologists and institutions leading to bias in the available evidence.^7 8^ Further weaknesses in the available evidence concern insufficient information on patient age, time to progression and mode of detection of future cancer.

Formulating a robust management strategy, when the published risk of malignancy of an atypia ranges widely, is challenging. Furthermore, the evidence on the type and severity of subsequent breast cancer is scarce. Only Hartmann *et al*^16^ described the subsequent cancers in any detail. However, this study from the USA only included 698 women with ADH and/or ALH, where detection and treatment regimens are significantly different to the UK. For both ADH and ALH there was a predominance of invasive ductal cancers; 69% were grade 2 or grade 3% and 25% were node positive. It is, therefore, difficult to know how conservative or aggressive management pathways should be for the different subgroups of atypia.

There is currently no accepted international consensus regarding the definitive management of atypia following their identification. This large-scale study of atypia from the Sloane cohort study using the English screening programme data will provide robust and generalisable evidence from which to design tailored management strategies for individual patients with atypia.

### Study objectives

1. To characterise atypia in terms of type, method of investigation and women’s demographics.
2. To determine breast cancer risk over time by type of atypia.
3. To characterise the nature of subsequent cancers detected, and their prognostic features.
4. To communicate results to clinicians and women.
5. To recommend changes to the NHSBSP quality standards.

## Methods and analysis

We will report the study according to the REporting of studies Conducted using Observational Routinely-collected health Data (RECORD) statement, an extension to the Strengthening the Reporting of Observational Studies in Epidemiology guidelines.^18^

### Data sources, cleaning, and linkage

The Sloane atypia project is a prospective cohort of atypia diagnosed through UK NHSBSP screening from April 2003 to the present.^19^ For this analysis, English screening units are included with radiology, histopathology, surgery and radiotherapy proformas. Data include age at diagnosis, mammographic features, biopsy method, histological features, surgical treatment, adjuvant treatment and vital status.

The data will be extracted and prepared by the Sloane Project team with patient identifiers removed before being uploaded for researchers based at the University of Warwick who will undertake the analysis and lead the publication and dissemination of findings. Data are entered onto the Sloane database at Public Health England, which has built-in integrity checks, which are run, along with extra validation checks, for each data request. Missing data items are subsequently completed as far as possible.

As part of the Sloane Project follow-up methodology, subsequent development of breast cancer has already been identified by matching women by NHS number and date of birth to the English Cancer Registry held by the National Cancer Registration and Analysis Service (NCRAS). The Sloane follow-up data are accessed via the internal Data Sharing Agreement between the NCRAS and the NHSBSP. The atypia cases will also be matched to the Mortality and Birth Information System to collect mortality data for censoring follow-up, and the Breast Screening Data Repository for information on invitation and attendance at subsequent screening mammography appointments. All linkage will have been done by the Sloane Project team prior to data being sent to Warwick University and no further linkage will be required. The full dataset will be transferred to researchers at Warwick University for an anticipated start date of analysis of 1 December 2021.

### Inclusion and exclusion criteria

All women identified as having epithelial atypia in the Sloane database will be included; this includes patients with ADH, LISN (both ALH and LCIS) and FEA. Women will be followed up until the earliest of: date of death, date of first further event (diagnosis of ductal carcinoma in situ (DCIS) and/or invasive breast cancer in either breast or metastatic breast cancer), or 31 March 2018. We will exclude: bilateral primary cases or the ‘best prognosis’ atypia of the bilateral primaries; patients where DCIS was present in addition to the atypia; pleomorphic LCIS (as these are managed akin to DCIS); those with an unknown type of atypia; cases not from England; and patients without follow-up until 31 December 2018.

### Outcomes

The primary outcome will be subsequent invasive breast cancer per 1000 women diagnosed with atypia at 3 years and 6 years following atypia diagnosis (1 year is a secondary outcome). Secondary outcomes include subsequent ipsilateral breast cancer; subsequent contralateral breast cancer; and nature of subsequent cancer (type, grade, size and nodal status).

### Analysis

We will provide descriptive statistics including numbers with each type of epithelial atypia (LISN (which includes lobular neoplasia (LN) in the Sloane database), ADH and FEA); years of follow-up; age when atypia was detected; screening round when atypia was detected; diagnostic pathway; treatment received for the atypia (including number receiving mastectomy, multiple operations, endocrine treatment). We will show graphically how diagnostic pathway and treatment received have changed over time and consider potential implications of this as a confounder for length of follow-up. We will also produce summary statistics of the outcomes, for the whole cohort and by type of atypia.

We will investigate the histological nature of cancers detected by providing tabular summaries showing total number of cancers detected for all atypia groups (splitting LISN into LCIS and ALH when possible) and by providing characteristics of those cancers. These characteristics will include grade of DCIS and/or invasive carcinoma, size of the cancer, number of involved nodes, Nottingham Prognostic Index (for invasive tumours if data allows), ipsilateral or contralateral presentation, and screen or symptomatic detection (if data allows). We will also report number of deaths from breast cancer and deaths from other causes.

The primary analysis is to ascertain the breast cancer rate in the years following screening in women with atypia. We will calculate the rate, with a 95% CI, at the 3-year and 6-year time points, after the first and second rounds of screening post atypia diagnosis. This will include interval cancers and cancers detected at screening. We will then compare the 3-year rate estimate and CI with the 3-year rate for the general population, which will be matched as closely as possible to the Sloane cohort. We will discuss issues related to the comparison of rates from different cohorts. This analysis will be repeated for three types of atypia (ADH, FEA and LISN) separately.

The secondary analyses will be in two parts. First, we will analyse the rate at 1 year similarly to the analysis of the 3-year and 6-year rates in the primary analysis. We will then describe the rates at all three time points by age at atypia diagnosis to see if there are any indications of different results for women of different ages. Second, we will consider the time since atypia diagnosis that a breast cancer diagnosis was made. We will calculate the rates of cancer detection, with 95% CIs, over the follow-up period for the whole cohort and for the 3 types of atypia, with corresponding risk tables featuring the number at risk, number of events and time to cancer (with 95% CIs). Death from a cause other than breast cancer will be included as a competing risk. Comparison of time to cancer detection between the types of atypia groups will be made with appropriate tests, and we will report the median/mean times to cancer. We will apply a stratified test to account for differences in age, if appropriate.

If there are sufficient events to make more complex analysis feasible, we will extend the analysis to estimate the probability of cancer diagnosis over the period of follow-up for each group by using flexible parametric modelling. This will allow adjustment for other observed variables, such as age, in a multivariable analysis. The models will be repeated to investigate: (1) ipsilateral versus contralateral cancer; (2) different diagnostic pathways; (3) radiological calcification versus other imaging features and (4) the subdivision of LISN into its two component parts, ALH and LCIS. Quantitative variables such as age will be retained as continuous variables wherever possible.

We will report the overall patterns of missing data, exploring, if appropriate, the sensitivity to missing data using multiple imputation methods. We will also report the predicted cancer probabilities from 1 to 6 years, to assist with decision making regarding screening interval ranges post atypia diagnosis. We will also perform sensitivity analyses on whether a cancer diagnosed within 6 months of a diagnosis of atypia should be included or excluded (as probably representing cancers missed at screening).

When atypia is detected at baseline screening with narrow bore core biopsy (for example 14 gauge), extra tissue is subsequently taken either through vacuum-assisted biopsy (eg, 7 gauge) or open surgical biopsy. This choice could influence results if, for example, a more extensive surgical biopsy widely excised a precursor lesion. The probability of subsequent cancer detection may also be affected by the follow-up regimen (annual or triennial screening) as this will affect probability of detection. Annual screening after a diagnosis of atypia has been standard practice since 2016,^6^ but there was some variability prior to that. Biopsy methods and frequency of screening in the follow-up period will be considered as potential explanatory variables.

Since the aim of this project is to understand the probability of subsequent cancer in a representative cohort of women with screen detected atypia, we do not wish to adjust for other risk factors. For example, if presence of atypia was correlated with very high genetic risk of cancer, and within 3 years of atypia detection mammography detectable cancer develops but not directly resulting from the atypia, then policy-makers would wish to see that risk included in our results rather than modelled out through inclusion of genetics as a confounder. The predictors of cancer development are very complex, and our approach will not be to try to fully model these, but instead focus on the generalisability of the sample to screening practice to inform policy.

### Sample size

On the most recent data review (24 September 2020) the Sloane database had 3645 cases of epithelial atypia with between 1 and 15 years of follow-up. Of these, 3043 meet our study inclusion criteria from the English NHSBSP, of which 1530 are recorded as LN/LISN, 1068 as ADH and 445 FEA. There are 496 patients with histological details awaiting addition to the database. The number of individuals is larger than in any other previous primary study with comparable follow-up, a high level of data completion and information on follow-up and treatment. We expect less variability in the data than encountered in systematic reviews because of standardisation of reporting cases using proformas for data submission and because of in-built quality assurance measures in the NHSBSP, including pathology guidelines for diagnosis and reporting of lesions.^20^

Critical information for policy-makers is the proportion of women with each type of atypia who develop cancer in the 3 years prior to their next scheduled screen (and the type of cancer detected), to determine whether yearly screening is necessary or standard 3 yearly screening is sufficient.

We have estimated the rate of breast cancer in the general population at 1 year, 3 years and 6 years after screening. The 1-year rate uses interval cancer data only,^21^ and the 3-year rate includes screen detected cancers (from women invited to be screened again within 5 years of their previous screen) as well.^1^ The 6-year rates are estimates, produced by doubling the values at 3 years (see table 1).

**Table 1:** Estimates of the rate of breast cancer per 1000 women at 1, 3 and 6 years after screening

Year	1	3	6
Interval cancers per 1000 women^21^	0.553	2.907	5.814
Screen detected cancers per 1000 women^1^	–	8.007	16.014
Total	0.553	10.914	21.828

For the sample sizes given for each type of atypia we have calculated what the minimum observed rate (to one decimal place) would need to be for its 95% CI (Wilson, with continuity correction)^22^ to be above the general population rate. This was performed for the three types of atypia we are considering separately, and also combined (table 2). Table 3 shows the breast cancer rate in women with atypia that is detectable with 80% power in a two-sided 5% significance test comparing it to the breast cancer rate in the general population, for each type of atypia and for all types combined, at 1, 3 and 6 years after diagnosis with atypia.

**Table 2:** Observed breast cancer rate per 1000 women required for 95% CI to be above the general population rate

		Year 1		Year 3		Year 6	
	Cases	Rate	95% CI	Rate	95% CI	Rate	95% CI
Population rate		0.55		10.91		21.83	
Projected rate using existing evidence		2.2–100		44		87	
Lowest rate observable using our data, for which 95% CI wholly above population rate							
Flat epithelial atypia	445	3.9	(0.57 to 17.1)	21.7	(10.92 to 41.3)	36.6	(21.88 to 59.7)
Atypical ductal hyperplasia	1068	2.5	(0.58 to 8.5)	17.7	(10.98 to 28.1)	31.1	(21.86 to 43.8)
Lobular neoplasia/lobular in situ neoplasia	1530	2.1	(0.57 to 6.4)	16.5	(10.96 to 24.6)	29.5	(21.85 to 39.6)
All types	3043	1.6	(0.58 to 4.0)	14.8	(10.94 to 19.9)	27.2	(21.85 to 33.8)

**Table 3:** Atypia rate of breast cancer (per 1000 women) detectable at 80% power compared with the general population rate

	Cases	Year 1	Year 3	Year 6
Population rate		0.55	10.91	21.83
Projected rate using existing evidence		2.2–100	44	87
Minimum atypia rate detectable at 80% power				
Flat epithelial atypia	445	80.6	29	45.4
Atypical ductal hyperplasia	1068	44	21.6	36.1
Lobular neoplasia/lobular in situ neoplasia	1530	35.2	19.6	33.5
All types	3043	23.9	16.8	29.9

This level of precision is anticipated to be sufficient for policy making. The main analysis is at 3 years. Here, if the rate for all types of atypia is 14.8 per thousand or higher, we will be able to measure that with a 95% CI not overlapping the general population figure, so we could detect an increase of 3.9 per thousand women screened or more. When applied to the rarest type of atypia, FEA, if the rate is 21.7 per thousand or higher, we will be able to measure that with a 95% CI not overlapping the general population figure. Small case control studies have estimated that the risk of cancer may be four times higher in women with atypia, so around 44 per thousand at 3 years. We have many more cases than would be required to detect such a change, for atypia overall and for each subtype.

For the secondary outcome of cancer detection rate at 1 year after screening we are examining whether there is a very substantial increase, representing an issue with the biopsy procedure/decision making at the screening episode at which the diagnosis of atypia was made; in other words, whether it is likely that there was a missed cancer that was present contemporaneously. Such an issue may result in a very high rate of 20–100 per thousand women screened. With these sample sizes an observed rate of 3.9 per thousand or more would produce a 95% CI above the general population rate, even for the rarest form of atypia included.

### Bias and confounding

Women with atypia are not typically given any cancer treatment in the UK (chemotherapy, radiotherapy or hormonal therapy), so that will not bias cancer recurrence rates downwards. We will also consider, if possible, any effects due to the long period of time that women with atypia diagnoses have been added to the Sloane atypia database.

As the Sloane atypia database is not a consecutive or random selection of cases of epithelial atypia in England, but is reliant on centres voluntarily reporting cases, there is the potential for selection bias from two sources. First, if selection was associated with subsequent outcomes (development of cancer) this would represent serious bias. However, this is likely not the case, because data are prospectively collected before subsequent outcomes are known. Second, non-random selection of cases at the point of detection (eg, more interesting cases), would change the spectrum of disease included and limit generalisability. We expect the selection to be incomplete but will check this empirically. There are 924 cases from 30 centres who have provided a complete consecutive sample (checked through retrospective review), whose characteristics we will compare to the rest of the cohort from centres with incomplete reporting. We will also repeat all of the main analyses just for the subset of cases from centres with complete reporting. Since 2014/2015, a list of numerical identifiers for women with atypia are identified by computer report, and centres work through the cases systematically according to administrative capacity, greatly limiting selection bias.

Variables such as comorbidities, fitness levels, socioeconomic status, family history and genetic testing are not collected as part of the Sloane project and are not collected as part of the NHSBSP; and are, therefore, not available for this study.

Validation of atypia data has been performed by cross checking with original screening unit source documents for patients with recurrence, and more generally against the Association of Breast Surgery national audits (ABS/NHSBSP) 2006/2007–2011/2012.^23^ Missing (unknown) data are rare in the Sloane audit for key comparisons, including use of radiotherapy (0.5%), grade (0.1%), tumour size (0.4%) or cause of death (0.1%).

Central pathology review is not performed in the Sloane audits, and there may therefore be inconsistencies in the diagnosis of epithelial atypia; for example, over the period of the audit, and histopathology terminology has changed regarding classification of FEA. However, the NHSBSP Pathology QA programme provides some mitigation for standardisation of diagnosis across units but not for changes over time.

Finally, lesions may have multiple types of atypia present in the same biopsy and different processes may also be seen in the core biopsy and subsequent vacuum-assisted or surgical biopsy. This will be considered carefully in terms of internal validity and generalisability when drawing conclusions from the research and adjusted for as part of the analysis where possible.

### Patient and public involvement

Warwick Medical School and the Sloane Project both have a longstanding partnership with the Independent Cancer Patients’ Voice (ICPV) to include patient representation at every stage of research. The Sloane Atypia Audit Project has been developed in partnership with the ICPV. The variability of care between screening centres has been identified as a priority throughout this time, and our proposed analysis followed by development of national guidelines is designed to reduce this variability.

The PPI advisors regularly meet with the members of the Sloane project to discuss progress, and comment from a patient point of view on research ideas, clinician surveys and communication. They have contributed to the development of the outline research application and have commented on this study protocol. Particular tasks will include:

- Assisting with interpretation of study findings, considering their knowledge and experience.
- Coproducing documents summarising study findings (eg, plain English summaries), and website content aimed at dissemination to the public.
- Advising on the communication of study findings to both the breast screening community and the public.
- Assisting in leading two focus groups in which we will explain our findings and explore recommendations based on these findings, to receive different perspectives on our conclusions, and identify other implications of our proposed changes for women screened.

Further, the wider ICPV community, and representatives of the public, will be involved in reviewing website content aimed at public dissemination of results.

## Ethics and dissemination

We have received research ethics approval from the University of Warwick Biomedical and Scientific Research Ethics Committee (BSREC 10/20–21, 8 October 2020), Public Health England office for data release approvals (ODR1718_313) and approval from the English Breast Research Advisory Committee (BSPRAC_031).

We will communicate findings to breast screening clinicians through journal publications and relevant conferences. We will also communicate results directly to breast cancer charities, including Breast Cancer Now and Cancer Research UK and to the general public, particularly women of screening age.

We will recommend changes to the NHSBSP Quality Assurance Guidance through holding two workshops with policy-makers and coauthors. Updating this guidance is key to implementation because this guidance is used as the basis for commissioning breast screening services.

## Discussion

We present the protocol for the first analysis of the Sloane atypia database to establish cancer rates over time by atypia type in women diagnosed with atypia through routine breast cancer screening in England. Current evidence is scanty and/or may not be generalisable to the UK breast screening programme. Our study has several strengths, including, a large prospective cohort from April 2003 to the present with long follow-up which allows the detection of subsequent cancers. The Sloane database has standardised reporting and high completion. However, undertaking an observational analysis does not allow the systematic inclusion or random selection of all women diagnosed with atypia at screening and has the disadvantage of introducing risk of bias from measured and unmeasured confounding, which will be addressed by repeating some analyses on the data from the centres that have complete cases only for comparison. This large-scale study of atypia from the English screening programme will help to address the current knowledge gaps to enable policy-makers to design tailored management strategies for individual atypia.

## Supplementary Material

Reviewer comments

## References

[1] Screening & Immunisations Team NHS Digital. Breast screening programme England, 2018-19, 2020. Available: https://files.digital.nhs.uk/0A/9D9F34/breast-screening-programme-eng-2018-19-report.pdf [Accessed 19 Feb 2020].

[2] Marmot MG, Altman DG, Cameron DA, et al. The benefits and harms of breast cancer screening: an independent review. Br J Cancer 2013;108:2205–40. 10.1038/bjc.2013.177 23744281 PMC3693450

[3] Said SM, Visscher DW, Nassar A, et al. Flat epithelial atypia and risk of breast cancer: a Mayo cohort study. Cancer 2015;121:1548–55. 10.1002/cncr.29243 25639678 PMC4424157

[4] Andreu FJ, Sáez A, Sentís M, et al. Breast core biopsy reporting categories—An internal validation in a series of 3054 consecutive lesions. Breast 2007;16:94–101. 10.1016/j.breast.2006.06.009 16982194

[5] Forester ND, Lowes S, Mitchell E, et al. High risk (B3) breast lesions: what is the incidence of malignancy for individual lesion subtypes? A systematic review and meta-analysis. Eur J Surg Oncol 2019;45:519–27. 10.1016/j.ejso.2018.12.008 30579653

[6] Public Health England. NHS breast screening programme: clinical guidance for breast cancer screening assessment, 2016. Available: https://assets.publishing.service.gov.uk/government/uploads/system/uploads/attachment_data/file/567600/Clinical_guidance_for_breast__cancer_screening__assessment_Nov_2016.pdf [Accessed 19 Feb 2020].

[7] El-Sayed ME, Rakha EA, Reed J, et al. Predictive value of needle core biopsy diagnoses of lesions of uncertain malignant potential (B3) in abnormalities detected by mammographic screening. Histopathology 2008;53:650–7. 10.1111/j.1365-2559.2008.03158.x 19076681

[8] Dyrstad SW, Yan Y, Fowler AM, et al. Breast cancer risk associated with benign breast disease: systematic review and meta-analysis. Breast Cancer Res Treat 2015;149:569–75. 10.1007/s10549-014-3254-6 25636589

[9] King TA, Pilewskie M, Muhsen S, et al. Lobular carcinoma in situ: a 29-year longitudinal experience evaluating clinicopathologic features and breast cancer risk. J Clin Oncol 2015;33:3945–52. 10.1200/JCO.2015.61.4743 26371145 PMC4934644

[10] Page DL, Dupont WD, Rogers LW, et al. Atypical hyperplastic lesions of the female breast. A long-term follow-up study. Cancer 1985;55:2698–708. 10.1002/1097-0142(19850601)55:11&lt;2698::AID-CNCR2820551127&gt;3.0.CO;2-A 2986821

[11] Zhou W-B, Xue D-Q, Liu X-A, et al. The influence of family history and histological stratification on breast cancer risk in women with benign breast disease: a meta-analysis. J Cancer Res Clin Oncol 2011;137:1053–60. 10.1007/s00432-011-0979-z 21499874 PMC3112325

[12] Shah-Khan MG, Geiger XJ, Reynolds C, et al. Long-term follow-up of lobular neoplasia (atypical lobular hyperplasia/lobular carcinoma in situ) diagnosed on core needle biopsy. Ann Surg Oncol 2012;19:3131–8. 10.1245/s10434-012-2534-9 22847124

[13] Mao K, Yang Y, Wu W, et al. Risk of second breast cancers after lobular carcinoma in situ according to hormone receptor status. PLoS One 2017;12:e0176417. 10.1371/journal.pone.0176417 28467490 PMC5415001

[14] Collins LC, Aroner SA, Connolly JL, et al. Breast cancer risk by extent and type of atypical hyperplasia: an update from the nurses' health studies. Cancer 2016;122:515–20. 10.1002/cncr.29775 26565738 PMC4742394

[15] Castells X, Domingo L, Corominas JM, et al. Breast cancer risk after diagnosis by screening mammography of nonproliferative or proliferative benign breast disease: a study from a population-based screening program. Breast Cancer Res Treat 2015;149:237–44. 10.1007/s10549-014-3208-z 25503778 PMC4298666

[16] Hartmann LC, Degnim AC, Santen RJ, et al. Atypical hyperplasia of the breast — risk assessment and management options. N Engl J Med Overseas Ed 2015;372:78–89. 10.1056/NEJMsr1407164 PMC4347900 25551530

[17] Page DL, Schuyler PA, Dupont WD, et al. Atypical lobular hyperplasia as a unilateral predictor of breast cancer risk: a retrospective cohort study. Lancet 2003;361:125–9. 10.1016/S0140-6736(03)12230-1 12531579

[18] Benchimol EI, Smeeth L, Guttmann A, et al. The reporting of studies conducted using observational routinely-collected health data (RECORD) statement. PLoS Med 2015;12:e1001885. 10.1371/journal.pmed.1001885 26440803 PMC4595218

[19] Public Health England. Breast screening: the Sloane project. Available: http://www.gov.uk/phe/sloane-project [Accessed 12 May 2020].

[20] The Royal College of Pathologists. Guidelines for non-operative diagnostic procedures and reporting in breast cancer screening, 2021. Available: https://www.rcpath.org/uploads/assets/4b16f19c-f7bd-456c-b212f557f8040f66/G150-Non-op-reporting-breast-cancer-screening.pdf [Accessed 16 Sep 2021].

[21] Bennett RL, Sellars SJ, Moss SM. Interval cancers in the NHS breast cancer screening programme in England, Wales and Northern Ireland. Br J Cancer 2011;104:571–7. 10.1038/bjc.2011.3 21285989 PMC3049599

[22] Newcombe RG. Two-sided confidence intervals for the single proportion: comparison of seven methods. Stat Med 1998;17:857–72. 10.1002/(SICI)1097-0258(19980430)17:8&lt;857::AID-SIM777&gt;3.0.CO;2-E 9595616

[23] Association of Breast Surgery. NHS BSP & ABS audit of screen detected breast cancer. Available: https://associationofbreastsurgery.org.uk/professionals/audit/nhs-breast-screening-programme-audit/ [Accessed 9 Sep 2021].

