# Supplementary material for: Breast screening atypia and subsequent development of cancer: protocol for an observational analysis of the Sloane database in England (Sloane atypia cohort study)
Source: BMJ Open. 2022 Jan 7;12(1):e058050. doi: 10.1136/bmjopen-2021-058050 (PMC8744119; doi:10.1136/bmjopen-2021-058050)
Supplement: Reviewer comments [file bmjopen-2021-058050.reviewer_comments.pdf]

PEER REVIEW HISTORY

BMJ Open publishes all reviews undertaken for accepted manuscripts. Reviewers are asked to complete a checklist review form (**http://bmjopen.bmj.com/site/about/resources/checklist.pdf**) and are provided with free text boxes to elaborate on their assessment. These free text comments are reproduced below.

ARTICLE DETAILS

**TITLE (PROVISIONAL)**	Breast screening atypia and subsequent development of cancer: protocol for an observational analysis of the Sloane database in England (Sloane atypia audit)
**AUTHORS**	Jenkinson, David; Freeman, Karoline; Clements, Karen; Hilton, Bridget; Dulson-Cox, Joanne; Kearins, Olive; Stallard, Nigel; Wallis, M. G.; Sharma, Nisha; Pinder, Sarah; Provenzano, Elena; Stobart, Hilary; McDonnell, Samantha; Taylor-Phillips, Sian

This article was not externally reviewed at BMJ Open. Protocols that have been independently assessed prior to submission to BMJ Open are usually fast-tracked to publication on the grounds that further substantial changes will not be possible. This independent assessment will usually be external, independent review for both a substantial grant award from a non-commercial or government funder and ethics approval.
